# Perioperative stroke: A perspective on challenges and opportunities for experimental treatment and diagnostic strategies

**DOI:** 10.1111/cns.13816

**Published:** 2022-02-27

**Authors:** Xia Jin, Peiying Li, Dominik Michalski, Shen Li, Yueman Zhang, Jukka Jolkkonen, Lili Cui, Nadine Didwischus, Wei Xuan, Johannes Boltze

**Affiliations:** ^1^ Department of Anesthesiology Renji Hospital School of Medicine Shanghai Jiaotong University Shanghai China; ^2^ Department of Neurology University of Leipzig Leipzig Germany; ^3^ Department of Neurology and Psychiatry Beijing Shijitan Hospital Capital Medical University Beijing China; ^4^ Department of Neurology and A. I. Virtanen Institute for Molecular Sciences University of Eastern Finland Kuopio Finland; ^5^ Department of Neurology Xuanwu Hospital Capital Medical University Beijing China; ^6^ School of Life Sciences University of Warwick Coventry UK; ^7^ Department of Radiology University of Pittsburgh Pittsburgh USA; ^8^ McGowan Institute for Regenerative Medicine University of Pittsburgh Pittsburgh USA

**Keywords:** experimental treatment, neuroprotection, perioperative stroke, stroke, translational research

## Abstract

Perioperative stroke is an ischemic or hemorrhagic cerebral event during or up to 30 days after surgery. It is a feared condition due to a relatively high incidence, difficulties in timely detection, and unfavorable outcome compared to spontaneously occurring stroke. Recent preclinical data suggest that specific pathophysiological mechanisms such as aggravated neuroinflammation contribute to the detrimental impact of perioperative stroke. Conventional treatment options are limited in the perioperative setting due to difficult diagnosis and medications affecting coagulation in may cases. On the contrary, the chance to anticipate cerebrovascular events at the time of surgery may pave the way for prevention strategies. This review provides an overview on perioperative stroke incidence, related problems, and underlying pathophysiological mechanisms. Based on this analysis, we assess experimental stroke treatments including neuroprotective approaches, cell therapies, and conditioning medicine strategies regarding their potential use in perioperative stroke. Interestingly, the specific aspects of perioperative stroke might enable a more effective application of experimental treatment strategies such as classical neuroprotection whereas others including cell therapies may be of limited use. We also discuss experimental diagnostic options for perioperative stroke augmenting classical clinical and imaging stroke diagnosis. While some experimental stroke treatments may have specific advantages in perioperative stroke, the paucity of established guidelines or multicenter clinical research initiatives currently limits their thorough investigation.

## Graphical abstract

Perioperative stroke is a feared condition due to difficulties in timely detection and overall worse outcome compared to spontaneously stroke. Experimental treatment strategies may provide specific benefits when targeting perioperative stroke and have been assessed regarding their applicability and feasibility in perioperative stroke.

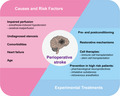

## 1 INTRODUCTION

According to the Society for Neuroscience in Anesthesiology and Critical Care (SNACC) definitions, perioperative stroke (PS) describes an ischemic or hemorrhagic cerebral event during or up to 30 days after surgery.^1^ Non‐modifiable risk factors for PS are higher age and female sex.^2^ The reasons for the higher PS risk in female patients are not well understood, but it is supposed that it may be related to faster progression of atherosclerosis after menopause.^2^ PS is most frequent after cardiac and neurosurgery, as well as after vascular thoracic and transplantation surgeries.^3^ The main etiology of ischemic PS seems to be embolic, but perfusion‐related events are also of relevance. For instance, severe anesthesia‐induced hypotension is associated with PS or can contribute to cerebral mal‐perfusion in intra‐interventional PS.^4^ Previously undiagnosed stenosis of a large brain‐supplying artery may also play a role. The vast majority of PS, about 95%, is of ischemic nature.^5^, ^6^ Consequently, this review will focus on ischemic events.

Perioperative stroke is a feared condition for numerous reasons. First, it is relatively frequent. In‐depth analysis of more than 520,000 cases reported an overall incidence of 0.1% in patients undergoing non‐cardiac surgery, which increased to 1.9% in high‐risk populations.^7^ A large‐scale retrospective investigation based on the US National Inpatient Sample involving over 10.5 million cases revealed a PS rate of 0.52% in patients undergoing non‐cardiac surgery in 2004. Interestingly, the rate increased to 0.77% in 2013.^8^ Although these relative numbers may seem moderate, the large amounts of surgeries performed each year result in a considerable number of PS cases. Moreover, PS can be much more frequent in some populations of surgical patients, especially those who undergo cardiac surgery. In detail, PS rates of 8.8% were reported for mitral valve surgery, and up to 9.7% for double or triple valve surgery, respectively.^9^

Second, PS can appear as a clinically silent event, sometimes referred to as covert PS, which is unrecognized on onset but detected later, for instance by means of brain imaging. Reasons for covert PS can be minor, subtle, or not properly classified symptoms. Moreover, the patient may be anesthetized or sedated during PS onset thus does not present obvious clinical symptoms. A recent multicenter clinical study revealed that covert PS appeared in 1 out of 14 (7.1%) non‐cardiac surgical patients aged 65 or older. PS was also associated with a higher risk for cognitive decline 1 year after surgery.^10^

Third, outcome after PS tends to be worse than in non‐surgical (NS) stroke patients. In detail, mortality rates in PS patients can be up to eightfold higher than in comparable NS populations, with mortality rates of up to 26%.^11^, ^12^ This illustrates the additional burden that PS inflicts on patients, their relatives, caretakers, and healthcare systems.

Common clinical reasons for the unfavorable outcome after PS are delayed diagnosis and intervention in case of covert PS, and potential difficulties in applying recanalization treatments (thrombolysis and thrombectomy) due to the recent surgery, medications that affect the coagulation system or the general condition of the patient.^13^ Recent SNACC guidelines recommend that early endovascular thrombectomy should be considered in PS patients to restore the cerebral blood flow.^14^ However, this therapeutic strategy requires clinical detection of the ischemic event and cerebral imaging including visualization of cerebral arteries in a narrow time window. PS patients also have an increased risk of hemorrhage independent of the cerebral event, which limits the use of intravenous thrombolysis markedly.^15^, ^16^ The patients' general condition may further complicate early initiations of rehabilitative actions.

This complex clinical situation warrants assessment of experimental treatment options that are currently under development to augment established therapeutic strategies for stroke, regarding their potential applicability, limitations, and benefits in PS. This includes consideration of potential PS‐specific pathophysiological mechanisms, related to the surgery or the underlying condition requiring it, which may contribute to the unfavorable PS outcome.

## 2 DIFFERENCES IN PATHOPHYSIOLOGICAL MECHANISMS IN PERIOPERATIVE VERSUS SPONTANEOUSLY OCCURRING STROKE

Clinical and logistical necessities affecting PS management and contributing to the unfavorable PS outcome are well known. However, basic pathophysiological reasons for the unfavorable outcome are incompletely understood and subject to current research. A general limitation of these studies is that a specific animal model for PS does not exist. Instead, models of surgical interventions are combined with commonly used approaches to induce focal cerebral ischemia in an experimental subject. Nevertheless, preclinical studies using these PS models provide preliminary evidence for important pathophysiological differences between PS and NS stroke that may substantially contribute to inferior PS outcome.^7^, ^17^ For instance, preclinical data suggest that the relatively high PS incidence in elderly and comorbid patients could be related to atherosclerotic plaque instability caused by perioperative stress.^18^, ^19^ Plaque volume, stability, and signs for plaque rupture were investigated in apolipoprotein‐E‐deficient mice. Mice were fed a high cholesterol diet, inducing a well‐accepted model to investigate plaque vulnerability.^20^ Some animals underwent laparotomy plus a major (about 20%) blood withdrawal, determined as the double hit paradigm.^21^ Plaque volumes were significantly increased in the double hit group, and more plaques were classified as bearing signs of rupture 3 days after inducing perioperative stress. Surgery or blood loss alone also increased plaque volumes, but inter‐group differences were not statistically significant. Treatment with 80 mg/kg atorvastatin starting 3 days prior to surgery reduced cholesterol as well as plaque volume and instability.^21^

Bone fracture models simulate a condition requiring surgical intervention and the surgical procedure itself. A surgically induced tibia fracture 6 or 24 h prior to experimental stroke by permanent middle cerebral artery occlusion (pMCAO) in mice increased lesion volumes and impaired functional outcome. Injury was most severe when pMCAO was induced 6 h after bone fracture.^22^ There were increased numbers of microglia and bone marrow‐derived macrophages around the ischemic lesion in mice with tibia fracture, indicating increased inflammation.^23^, ^24^ Moreover, there was a pronounced blood–brain barrier (BBB) breakdown.^25^ Detrimental effects were also seen when bone fracture was induced after pMCAO.^26^ Post‐stroke bone fractures are further associated with larger brain edema volumes in subacute stroke stages,^27^ potentially due to mentioned BBB breakdown. The detrimental effects of bone fracture on stroke outcome are partially reversed by α‐7 nicotinic acetylcholine receptor agonists diminishing neuroinflammation and promoting pro‐regenerative mechanisms.^28^, ^29^

These preclinical results strongly indicate that aggravated (neuro‐)inflammatory reactions, promoted by pre‐ or post‐stroke bone fracture and surgery, strongly contribute to unfavorable PS outcome. However, the exact mechanisms have not been fully clarified. Importantly, comorbidities frequently seen in elderly or surgically stressed patients such as hypertension can further aggravate innate immune and inflammatory reactions,^30^ suggesting a potential relevance in PS.

## 3 CHALLENGES AND OPPORTUNITIES FOR EXPERIMENTAL TREATMENT STRATEGIES IN PS

Once proven safe and effective, experimental treatment strategies for stroke may help to counter PS. The idea is supported by the fact that many experimental stroke treatment strategies target neuroinflammation which seems to play a prominent pathophysiological role in PS.^31^ In general, experimental treatments should be compatible to recanalization procedures, but may also be applied in PS patients being ineligible for recanalization.^15^, ^16^ However, feasibility, safety, and potential efficacy of experimental treatments must be reviewed considering the particular clinical circumstances in which they would be applied for PS. In this context, it is important to consider that about half of all PS occur within 24 h and 93% within 72 h after surgery.^11^ Table 1 provides an overview on potential experimental treatment that may be feasible for PS.

**TABLE 1:** Overview on potential experimental therapeutics for perioperative stroke

Approach	Advantages	Disadvantages	Safety	Applicability
Pharmacological neuroprotection	Already clinical data in ischemic stroke, could be applied preventively	Most approaches only address one mechanism, efficacy still unclear	+	++
Inhalative substances	Easy applicability and good control, good effect described for iNO and argon	Some safety concerns with inhalation anesthetics and long‐term iNO application, relatively high costs	+/‐ to ++	+/‐ to +
Intravenous anesthetics	Easy applicability, excellent safety profile	Overall effects unknown and moderate at best	++	+
Cell therapies, stem cells	Potentially address multiple mechanisms	Overall effects unclear, safety concerns for some applications, limited application scenarios	+/‐	+/‐
Cell therapies, immune cells	Potentially address multiple mechanisms, good applicability	Overall safety and effects still unclear, still experimental (no clinical data) autologous approaches require ex vivo cell processing	?	+
Pre‐ and postconditioning	Easy applicability, multiple effects, potentially wide therapeutic time window, inexpensive	Still experimental	++	++
Restorative approaches	Long, potentially unlimited therapeutic time window	Still experimental, overall safety and efficacy unclear	?	‐

Abbreviations: ++, very good; +, good; +/‐, moderate; ‐, not optimal; ?, unclear; iNO, inhalative nitric oxide.

### 3.1 Pharmacological neuroprotection

Recent advances in recanalization therapies, especially by mechanical thrombectomy, provide novel opportunities for the restoration of the cerebral blood supply and thus for additional pharmacological neuroprotection.^32^, ^33^ Combining pharmacological neuroprotection with recanalization enables delivery of neuroprotectants exactly where and when needed. Next‐generation neuroprotectants such as nerinetide, a postsynaptic density protein 95 inhibitor counteracting oxidative stress, are clinically tested in combination with recanalization therapies.^34^ The advantage of spatially and temporally targeted neuroprotectant delivery only comes to effect for those patients who can benefit from recanalization. However, recanalizing strategies such as mechanical thrombectomy are still available to the minority of patients with spontaneously occurring stroke, primarily due to a lack of salvageable brain tissue, contraindications, or lack of access to centers performing these techniques.^35^ PS patients may experience additional therapeutic restrictions due to late diagnosis (covert PS), their general condition, or specific medications impeding the use of intravenous thrombolysis and partly mechanical thrombectomy. However, these patients would still be eligible for pharmacological neuroprotection as a stand‐alone treatment.

For instance, targeting neuroinflammation pathways is a potential neuroprotective strategy for treating ischemic stroke. Salmeron et al. unexpectedly found that the pro‐inflammatory cytokine interleukin (IL)‐1α exerts therapeutic effects after stroke by promoting proangiogenic and neurogenic mechanisms, potentially outweighing its pro‐inflammatory effects.^36^ Moreover, Franke et al. demonstrated that the blockade of inflammasome NLR Family Pyrin Domain Containing 3 (NLRP3) molecular protein complexes, which initiate inflammatory responses, limits inflammatory‐driven infarct growth during transient (t)MCAO and after recanalization in mice.^37^

On the contrary, there is a long history of neutral and negative efficacy trials of stand‐alone neuroprotection in the clinic.^38^ In addition to quality limitations in some preclinical studies, the previous failure of pharmacological neuroprotection emerged from design differences in preclinical versus clinical trials.^39^ For instance, many pharmacological neuroprotectants are most effective when given shortly after stroke onset. This also accounts for nerinetide, which was most effective when given 1.5 h after stroke onset in primates.^40^ These narrow time windows are on the lower end of what is achievable in a clinical scenario and seem unrealistic in the context of covert stroke. Analyses from the Highly Effective Reperfusion Using Multiple Endovascular Devices (HERMES) group revealed that time to treatment onset with alteplase was 74–140 min under ideal conditions.^41^ Some neuroprotectants even show best efficacy when given prior to stroke onset, what is not realistic in a scenario of spontaneously occurring stroke, but might represent a reasonable strategy for surgical interventions with high risk for PS.

Most surgeries are elective, and clinical investigation may help to identify vulnerable patients prior to PS based on their risk factor profile or the type of surgery they will undergo. Basically, risk factors like heart failure and significant carotid stenoses that may significantly affect brain perfusion during surgery are of special interest in this context. Further, pharmacological neuroprotection can be initiated pre‐surgically and maintained throughout all critical surgical and postsurgical periods. Combination of different neuroprotectants might be applied to cover multiple stroke pathomechanisms. This approach may not prevent PS per se, but potentially alleviate ischemic damage.^42^, ^43^ A single study reported the application of edaravone, a free radical scavenger which is used in Japan since 2001, in hypoxic–ischemic brain injury caused by unilateral common carotid artery occlusion plus hypoxia (7.5% O_2_/92.5% N_2_ applied via face mask) in mice. Prophylactic, that is, 30 min prior to the hypoxic–ischemic injury, but not delayed edaravone application mitigated ischemic tissue damage and improved functional outcome.^42^ Despite these encouraging findings, we still lack profound proof of concept for the perioperative neuroprotection paradigm, and future preclinical research is needed to explore potential therapeutic opportunities.

Another interesting option is metabolic support of brain tissue at risk for infarction. This paradigm would neither prevent PS nor long‐term tissue damage. However, it may decelerate penumbra decline and thus increase the time available for recanalizing interventions. Options comprise the pharmacological increase of collateral perfusion^44^, ^45^ or replenishment of energy‐containing substrates such as adenosine triphosphate.^46^ Although additional preclinical investigations are required to further assess and optimize the metabolic support paradigm particularly in an experimental PS setting, it was already applied successfully in aged animals and subjects expressing comorbidities similar to those of patients at risk for PS.^46^ Although successful, therapeutic effects are moderate and best only if the metabolic support is started relatively early after stroke onset. The approach could be initiated prior to surgical interventions in patients characterized by a relevant risk for PS. It might particularly benefit those in whom PS is not caused by a major embolic event blocking blood flow to a certain brain area, but primarily on cerebral hypoperfusion during surgery with a residual blood flow to the affected area.

Notably, specific agents may also extend the time window for vascular recanalization and further neuroprotectant administration. Tian et al. found that fingolimod, a drug typically used in the field of multiple sclerosis, may increase the efficacy of alteplase administration in the 4.5–6‐h time window by enhancing both anterograde reperfusion and retrograde collateral flow.^47^ This prospective clinical trial not only utilized an effective strategy to improve alteplase application in stroke patients who were ineligible for mechanical thrombectomy, but also points at the possibility to widen the time window. This could be beneficial in case of delayed PS detection.

In summary, pharmacological neuroprotection represents a promising strategy in the setting of PS based on the unique fact that initiating neuroprotection is possible prior to the ischemic event.

### 3.2 Inhalative substances

Some frequently used volatile anesthetics and other gaseous compounds are reported to have neuroprotective capabilities, which might be particularly valuable in a perioperative setting. Isoflurane and sevoflurane exposure prior to, during, and after cerebral ischemia was shown to reduce brain damage in rodents.^48^, ^49^ Therapeutic effects include a reduction in lesion volume, improvement of functional outcome,^50^ attenuation of reactive astrogliosis,^51^ and promotion of anti‐inflammatory microglial and macrophage phenotypes.^52^ Research revealed numerous molecular pathways that mediate the beneficial effects of volatile anesthetics.

There is strong evidence for isoflurane‐induced neuroprotection. Ischemic damage is inversely related to duration and dose of isoflurane anesthesia in tMCAO.^53^ Neuroprotective effects persisted at least 8 weeks after focal cerebral ischemia in rats.^48^ These effects are so strong that they may even mask the therapeutic benefits of potential neuroprotective drugs in stroke models.^54^ Moreover, volatile anesthetics including isoflurane show a dose‐dependent protective effect on the incidence and severity of early postoperative ischemic stroke in patients undergoing non‐cardiac surgery.^55^

On the contrary, both isoflurane and sevoflurane also exert detrimental effects on cognitive function in fetal and aged brains both in experimental models and patients^56^, ^57^, ^58^ although their impact is still controversially discussed for healthy adults.^59^ Both agents are also believed to induce inflammation‐mediated neurotoxicity in the young adult and aged brain.^60^, ^61^ Isoflurane also opens the blood–brain barrier.^62^ Moreover, anesthesia is often related to hypotension what is critical in the context of ischemic stroke.

Interestingly, neurotoxic effects predominantly emerge in the absence of brain injury, while neuroprotective effects are observed in cases of ischemic or traumatic brain injury.^63^ A potential reason is the reduction in neuronal activity and metabolism during anesthesia,^64^ which indeed would be beneficial in stroke but not in the steady state. Although this does not prevent the use of volatile anesthetics in patients at risk for PS, it does not suggest them as first‐line stand‐alone therapeutics. When considering a therapeutic application, sevoflurane might have the advantage of better applicability and a favorable safety profile in clinical settings.^65^ However, its application deserves detailed experimental investigation in perioperative setups including a thorough assessment of both protective and potential toxic effects to define its value for mitigating PS damage.

Normobaric and hyperbaric oxygenation have been discussed as potential neuroprotective approaches for decades. Currently, normobaric oxygenation is revisited.^66^ Meta‐analysis indicates a potential benefit of the approach,^67^ but large‐scale multicenter clinical trials are needed to assess this potential benefit. The situation is comparable for hyperbaric oxygenation.^68^ Results of these ongoing assessments in spontaneous stroke should be awaited before discussing a potential application in PS.

Profound neuroprotective effects were also shown for other substances such as inhalational nitric oxide (iNO). iNO dilatates blood vessels in areas of low oxygen concentrations. This effect is clinically utilized since the 1990s, for instance in newborns with persistent hypertension due to pulmonary vasoconstriction.^69^ iNO also increases cerebral blood flow in the penumbra during acute stroke.^70^ However, long‐term application of iNO comes with drawbacks such as methemoglobin formation and may cause mild to moderate hypotension.^71^, ^72^ Enhanced NO levels in the neuronal compartment are toxic, but the relatively short half‐live of NO and its rapid binding to hemoglobin make iNO an unlikely cause of neurotoxicity, especially when applied at concentrations of 50 ppm or lower. These low concentrations also alleviate the risk of a relevant reduction in blood pressure and thus cerebral perfusion, what would be particularly dangerous in an intensive care or surgical setting, characterized by periods of hypotension and vasopressor support. The challenges related to long‐term iNO application currently exclude it as an option for PS prevention, but it could play a role in prolonging the therapeutic time window for recanalization therapies should PS be detected.

The application of hydrogen has been reported in experimental stroke. The primary mode of action seems to be antioxidation that counters cytotoxic effects of reactive oxygen species.^73^ Hydrogen also possesses strong anti‐inflammatory effects that can be beneficial after stroke. For instance, hydrogen can attenuate post‐stroke activation of microglia while shifting microglial polarization toward the anti‐inflammatory M2 type.^74^ Hydrogen also attenuates the increase of pro‐inflammatory M1 macrophages.^75^ Anti‐apoptotic effects of hydrogen have been reported,^76^ and it stabilizes the blood–brain barrier after stroke in hypertensive rats.^77^ Of note, hydrogen can also be applied intravenously after being enriched in saline. The procedure is safe in patients after ischemic stroke including those treated with tissue plasminogen activator.^78^ Hydrogen application seems to be safe even in higher concentrations although the expression of certain enzymes such as aspartate aminotransferase, alanine aminotransferase, and γ‐glutathione transferase can decline after exposure.^79^ Application of hydrogen to mitigate PS may be considered after defining the most effective way of administration (gaseous versus enriched in saline) and further confirming safety. An advantage may be its compatibility with recanalization approaches and effectiveness in comorbid individuals.

Noble gases such as xenon and argon can exert neuroprotective effects. Argon may be preferred over xenon as it is less sedative and shows robust beneficial effects in experimental stroke and global cerebral ischemia. Argon reduces ischemic lesion size and improves functional outcome after 2 h of tMCAO,^80^ and prolonged exposure of 24 h is safe in rodents.^81^ Argon application is also safe in hemorrhagic stroke. Preclinical data are missing on PS, and argon was not yet applied clinically for NS stroke or PS. However, it might be an interesting therapeutic candidate for both PS prevention and treatment even in patients being ineligible for recanalization therapies because it is even beneficial after pMCAO.^81^ When applied therapeutically, the need for timely PS diagnosis remains.

### 3.3 Intravenous anesthetics for neuroprotection

Propofol was found to exert neuroprotective effects after ischemic stroke in preclinic studies. In detail, Adembri et al. demonstrated that propofol reduced infarct size and preserved spontaneous activity in rats and that propofol at a clinically relevant concentration mediated these protective effects by attenuating swelling of neuronal mitochondria.^82^ More recently, Wang et al. infused propofol at reperfusion after tMCAO and found decreased infarct size and attenuated neurological deficits in the propofol treated group, in which neurotoxic aggregation of α‐synuclein was also reduced.^83^ However, these findings raised discussions as a randomized clinical trial with 66 patients undergoing intracranial aneurysm surgery could not reveal protective propofol effects on postoperative cognitive function.^84^

Ketamine and dexmedetomidine are other, frequently applied anesthetic drugs with potential neuroprotective effects. Ketamine has anti‐apoptotic and anti‐neuroinflammatory effects after brain damage and preserves cognitive function after major cardiac surgery in humans.^85^, ^86^, ^87^ Most recently, Xiong et al. showed that MCAO‐induced brain damage was significantly attenuated by administration of (R)‐ketamine, but not (S)‐ketamine. The effect was seen after treatment prior to or after MCAO.^88^ Dexmedetomidine is a selective α2‐adrenergic agonist that was tested as a neuroprotective agent in experimental stroke models. Dexmedetomidine at a dose of 3 μg/kg given subcutaneously 30 min before and 3, 12, 24, and 48 h after global ischemia in gerbils protected neurons in the hippocampal CA3 and dentate gyrus.^89^ Intravenous infusion of dexmedetomidine (9 μg/kg) during tMCAO decreased infarct volume by 40%, being more effective than the NMDA receptor antagonist CGS‐19755, although a minor increase in blood glucose and hypotension was observed.^90^ Despite these early yet promising results, dexmedetomidine was never tested in clinical neuroprotection trials. However, due to its broad sedative, anxiolytic, analgesic, sympatholytic, and stable hemodynamic profile, dexmedetomidine is now used as an adjuvant for premedication, especially in patients susceptible to preoperative and perioperative stress. More importantly, dexmedetomidine improves cognition after carotid endarterectomy,^91^ reduces postoperative delirium after joint replacement surgery,^92^ and attenuates postoperative disability after cranial surgery^93^ pointing at its potential neuroprotective properties in a perioperative setting. Dexmedetomidine also reduces inflammatory factor expression and enhanced brain‐derived neurotrophic factor (BDNF) levels.^91^ Moreover, dexmedetomidine may mediate neuroprotective effects by anti‐apoptosis and suppression of immune responses.^94^, ^95^, ^96^ Whether or not injection anesthetics might be beneficial in PS remains to be investigated preclinically before moving on to any clinical assessment. Potential detrimental effects of anesthesia‐induced hypotension as discussed for volatile anesthetics must also be considered.

### 3.4 Cell therapies

Immune and stem cell therapies (ICT and SCT, respectively) were extensively investigated in animal models of ischemic stroke and are considered a promising therapeutic option.^39^, ^97^, ^98^ In the field of ICTs, regulatory T‐cell therapy is an important approach. CD4^+^CD25^+^Foxp3^+^ regulatory T (Treg) cells exert neuroprotection after ischemic stroke by suppressing neuroinflammation.^99^, ^100^ Adoptive transfer of Tregs was shown to ameliorate BBB damage in the acute phase of stroke^101^ and accelerates neural stem cell propagation in the subsequent brain repair phase.^102^ Inhibitory signaling molecules such as cytotoxic T‐lymphocyte‐associated protein 4, programmed cell death‐ligand 1, and IL‐10 contribute to the Treg‐mediated attenuation of neuroinflammation after stroke.^103^, ^104^, ^105^ Tregs also promote neural stem cell proliferation by secreting IL‐10 and by suppressing astrogliosis.^106^, ^107^ Treg‐derived osteopontin promoted post‐stroke white matter repair and functional recovery by fostering regenerative capacities of microglia via integrin receptor‐mediated signaling.^104^ Consistently, Ito. et al. found that Tregs suppress astrogliosis and foster neurological recovery, and that these processes peak about 2 weeks after stroke.^108^ However, the therapeutic window for Treg transplantation is not known. Age^109^ and sex^110^, ^111^ can influence the potency of Tregs and also affect microglial function.^112^ Any beneficial effects of ICTs in NS stroke or PS remain to be clinically investigated.

Another option of cell therapy for NS stroke is SCT. First clinical trials are underway to assess SCT safety and efficacy in NS stroke.^113^, ^114^, ^115^, ^116^ Adult cell populations such as mesenchymal stem cells (MSCs) from bone marrow^117^, ^118^ or adipose tissue, as well as mononuclear cell populations from bone marrow or umbilical cord blood (containing a stem cell fraction), are predominantly applied due to their proper safety profile, good applicability in a clinical setting, and the option for autologous treatment preventing potential immunological consequences.^119^ Nevertheless, allogeneic cell products including neural and neuronal stem and progenitor cells are also investigated. Allogeneic populations provide the advantage of being readily available as an off‐the‐shelf product and can be modified a priori to increase therapeutic efficacy.

Adult SCTs exert their therapeutic effects by indirect, mainly paracrine (“bystander”) functions.^120^ Particularly, they promote angiogenesis,^121^, ^122^ modify and mitigate reactive astrogliosis, exert general neuroprotective effects,^123^ or increase endogenous progenitor cell proliferation in the brain.^124^ Some populations such as MSCs also exhibit strong immunomodulatory and ‐suppressive abilities^125^, ^126^, ^127^ what might be useful given that aggravated neuroinflammation is an important PS pathomechanism.

However, SCTs may not be a first‐line treatment for PS, and application could be restricted to selected patient populations.^24^ A recent meta‐analysis showed that the clinical efficacy of adult SCTs in NS stroke currently stays behind therapeutic effects reported in preclinical studies. For instance, the bystander therapeutic effects of adult SCTs predominantly target pathomechanisms in acute and subacute stroke stages, meaning that their effective application has a therapeutic time window.^128^, ^129^ Although quality of preclinical stroke research, a known limiting factor for clinical translation in the past, has clearly improved,^130^ design differences between preclinical and clinical trials may contribute to minor efficacy seen in clinical trials.^131^ On the contrary, requirements of best practice stroke treatments often interfere with the application of SCTs, requiring an extension of the therapeutic time window of SCTs in clinical trials for logistical reasons. This, however, may significantly decrease SCT efficacy.^115^ Moreover, obtaining cells from autologous sources such as bone marrow or adipose tissue requires additional invasive procedures. This can be challenging, for instance in trauma patients undergoing surgery. Thus, SCTs for PS might be restricted to commercially available allogeneic cell products unless a previous “preventive” preservation of autologous cells was performed for the patient.

Finally, the application of adult cell populations, regardless of being autologous or allogeneic, is not free of risks. Improper intravenous or intra‐arterial administration of larger cells such as MSCs may cause pulmonary^132^ or cerebral embolism,^133^ respectively. Long‐term cryopreservation may further affect viability and subpopulation composition of adult cell populations.^129^ This may even reduce therapeutic efficacy in some populations.^134^ Extracellular vesicles (ECVs) might be a potential alternative. ECVs are released from cells upon fusion of the multivesicular body (an intermediate endocytic compartment) with the plasma membrane. ECVs are involved in inter‐cellular communication over long distances and contain messenger molecules and cytokines believed to mediate the therapeutic bystander effects reported for adult cell populations in stroke. Thus, ECVs derived from adult stem cell populations exert therapeutic effects similar to those of the cells.^135^ Moreover, they are much smaller and less immunogenic than allogeneic cells, avoiding many of the challenges associated with the use of adult cell populations. ECV application is also compatible with other conventional or experimental PS treatments as it only requires systemic bolus injection or infusion. However, there is no detailed knowledge on their efficacy in PS, and methods for ECV production have not yet reached an industrial scale and quality. Appropriate potency assays are also lacking.

### 3.5 Pre‐ and postconditioning strategies

Conditioning strategies are an emerging paradigm in experimental stroke research and already showed remarkable effects in other ischemic conditions such as myocardial infarction.^136^ Conditioning could be applied before (ischemic preconditioning; iPreC) or after the ischemic event (ischemic postconditioning; iPoC). The principle of iPreC is to expose the target organ (or a peripheral target) to one or multiple brief ischemic episodes. This induces tolerance against subsequent and more severe ischemic injuries, including in the brain.^137^ The effect is mediated by profound changes in cellular gene expression, for instance downregulating metabolism and immune responses.^138^ Similar to neuroprotection prior to the ischemic injury, iPreC may not play a major role in NS stroke, but would be applicable in patients at high risk for PS. Although the brain itself can be conditioned effectively in experimental subjects,^139^, ^140^ the clinically preferred approach would be remote iPreC, for instance in a limb (peripheral conditioning).

The therapeutic mechanisms of iPoC differ from those of iPreC. They include the mitigation of reperfusion injury^138^ and post‐stoke edema,^141^ BBB protection,^142^ and reduction in apoptosis.^143^ Some studies suggest that the therapeutic time window for remote iPoC may be surprisingly wide. Although a reduction in infarct volume was achieved only when applying iPoC within 24 h after 60 min tMCAO in mice, best functional recovery and even limited neuroregeneration was seen when iPoC started 5 days after tMCAO.^144^ Moreover, iPoC may extend the therapeutic time window for recanalization.^145^ Importantly, conditioning approaches are already tested clinically,^146^ and there is preliminary evidence for a modest therapeutic benefit in non‐human primates^147^ and humans.^110^ However, as in other fields, there are design differences between preclinical and clinical studies^148^ so final appraisal of the therapeutic impact is not yet possible.

There are also non‐ischemic conditioning strategies. For instance, a single pretreatment with isoflurane induces ischemic tolerance in the rat brain.^149^, ^150^ Multiple mechanisms are likely to be involved including reduced apoptosis via activation of the mitogen‐activated protein kinase (MAP) kinase ERK (p42/44) and protein kinase B (Akt).^151^ However, whether neuroprotection is persistent and effective in high‐risk patients remains open. Conditioning strategies appear as an easily applicable and safe option for PS prevention and therapy. Both iPreC and iPoC protocols could be applied in surgical patients, with some restrictions in trauma patients. Research is underway to unravel the exact mechanisms and mediators behind conditioning effects. Thus, the approach may turn into a pharmacological one in future, removing further hurdles for widespread application. Another advantage is that conditioning strategies can be applied reproducibly and at very low costs, not interfering much with standard care procedures even in an intensive care setting. However, neither preclinical nor clinical assessments of conditioning strategies were performed in PS so far. Moreover, preclinical research is warranted to optimize application, duration, and timing of conditioning strategies in PS before moving forward to early‐stage clinical trials.

### 3.6 Restorative approaches

Tissue restoration after stroke, regardless of occurring spontaneously or as PS, is currently thought to be challenging. The reason is a lack of anatomical, cellular, and molecular cues allowing the recapitulation of brain development as seen during ontogenesis.^120^ Biomaterials and scaffolds may help to provide such cues in larger lesions,^152^ whereas smaller lesions or those predominantly situated in the white matter may be attractive targets for restorative therapies.^25^, ^153^ However, a profound preclinical proof of concept of restorative approaches is not available so far, and any clinical application is unlikely to happen in the near future.

Another aspect of a potential brain repair is attracting increasing attention recently. Immune cell‐mediated long‐term neuroinflammation is an intriguing target for strategies trying to address or modulate various endogenous regenerative processes such as neurogenesis, white matter repair, and others.^154^

## 4 EXPERIMENTAL DIAGNOSTIC APPROACHES FOR CRITICALLY ILL POSTSURGICAL PATIENTS

A major challenge in PS management is its timely detection intra‐surgically or in the immediate postsurgical phase, and the clinical assessment is generally limited by the existing unconscious condition or reduced consciousness of the patient. Furthermore, postsurgical care, in particular for trauma patients, may require intensive anesthesia or even sedation in an intensive care setting, further complicating timely PS diagnosis. Brain imaging technologies such as magnetic resonance imaging and computed tomography are limited diagnostic options for the screening of PS, since they would need to be applied frequently.

An alternative method being under consideration for decades is electroencephalography (EEG) which has a high sensitivity in detecting pathological events in the brain. Difficulties in discriminating old versus new strokes, detecting strokes in sedated patients, and relatively low spatial resolution^155^ limit the use of EEG in PS. New data provide evidence that EEG might be a possible tool for the detection of stroke due to large artery occlusion,^156^ but this approach requires verification in further studies. Near‐infrared spectroscopy (NIRS) might be very sensitive in detecting cortical strokes. However, false‐negative results are possible due to a limited field of detection or in strokes located below the cortex.^157^, ^158^ Other technologies based on ultrasound^159^ and volumetric impedance phase‐shift spectroscopy^160^ are under development, but available data are not yet comprehensive enough for thorough validation of diagnostic feasibility in PS. An optimal diagnostic approach should also provide information on the location of the vessel occlusion which is essential for the individual consideration of recanalizing attempts.

Potential stroke biomarkers including purines,^161^ 5‐oxopoline,^162^ D‐dimers in combination with glial fibrillary acidic protein,^163^ or glutamate plus interleukin 6,^164^ S‐100β, neuron‐specific enolase, and matrix metalloproteinase‐9 were investigated as potential stroke biomarkers. All can be detected from blood samples. This allows continuous surveillance of patients at risk for PS. Although some approaches have a decent specificity and others such as broad metabolomic screening may discriminate stroke subtypes,^165^ a general problem of many biomarkers is a relatively low specificity. However, low specificity of biomarker‐based or other point‐of‐care stroke diagnostics is not necessarily a problem since reliable PS diagnosis may rely on a two‐step strategy. Highly sensitive and continuously applicable screening methods aiming to recognize any suspicious alteration as early as possible, and not interfering with gold standard care, would be applied in the first instance. In case of any abnormality being detected, conventional brain imaging to exclude PS or to decide about immediate therapeutic interventions is performed immediately. This two‐step strategy, in theory, may facilitate timely treatment of PS in critically ill patients. In practice, there would be limitations regarding the availability of the screening methods and trained staff to apply them reliably, as well as by costs incurring for continuous screening. Further clinical studies aiming to define the most feasible setup and confirming true therapeutic benefits will be required. Table 2 provides examples for potential stroke biomarkers which have shown promising results in clinical applications.

**TABLE 2:** Examples for potential stroke biomarkers

Marker	Study type	Patient details	Advantages	Disadvantages	Reference
Purines	Single center, prospective observational	18 patients undergoing carotid endarterectomy	Very sensitive, no complex sample processing, immediate results	Low specificity	^161^
D‐dimers +GFAP	Single center, retrospective observational	239 patients with suspected strokes in two (derivation, *n* = 128 and validation, *n* = 111) cohorts	High sensitivity and specificity, allows to safely triage large vessel occlusion patients (AUC = 93%)	Based on standard ELISA in its present form, thus time consuming and not yet eligible for screening	^163^
Glutamate +IL−6	Single center, retrospective	4775 stroke patients	High sensitivity and good specificity, ay indicate patients for recanalization	Diagnostic time up to 2 h in current setup, no data on hemorrhagic strokes yet	^164^
Asymmetrical and symmetrical dimethylarginine, pregnenolone sulfate, and adenosine	Single center, prospective	Patients with strokes (*n* = 508) and stroke mimics (*n* = 349), neurologically normal controls (*n* = 112)	High accuracy in discriminating ischemic stroke for stroke mimics	No data on hemorrhagic strokes yet, not sure whether diagnostic signature overlaps with other conditions	^165^

Abbreviations: AUC, area under the curve; ELISA, enzyme‐linked immunosorbent assay; GFAP, glial fibrillary acidic protein; IL, interleukin.

## 5 SUMMARY AND CONCLUSIONS

Perioperative stroke currently remains a feared condition due to a relatively high incidence and poor functional outcome. Established stroke treatments aiming at recanalization are of limited use in PS patients for several reasons including their narrow time windows and contraindications emerging from an increased bleeding risk. Experimental treatment strategies including peri‐surgical pharmacological neuroprotection, inhalative substances, as well as pre‐ and postconditioning approaches may improve PS prevention and treatment. However, we still lack profound translational and clinical data on safety and efficacy of these approaches, as well as continuously applicable diagnostic option for timely PS detection during surgery or in sedated patients. Collaborations between preclinical scientists, surgeons, neurologists, and anesthesiologists are required to improve PS management and outcome. Patients being at risk for PS would also benefit from internationally harmonized, evidence‐based consensus guidelines for PS diagnosis, management, and care.

## CONFLICT OF INTEREST

The authors declare that there are no competing interests.
